# Aging towards walkable futures: insights from a multidisciplinary workshop held in Barcelona, Spain

**DOI:** 10.1186/s12889-024-21223-z

**Published:** 2025-01-17

**Authors:** Enric Vall-Garcia, Laura Delgado-Ortiz, Lisa Alcock, Laura Coll-Planas, José Augusto García-Navarro, Susanne Iwarsson, Josep Maria Jansà, Carl-Philipp Jansen, Sarah Koch, Simon Schwartz, Merja Rantakokko, Adelaida Sarukhan, Willeke van Staalduinen, Lynn Rochester, Judith Garcia-Aymerich

**Affiliations:** 1 Barcelona Institute for Global Health (ISGlobal), Barcelona, Spain; 2 Universitat Pompeu Fabra (UPF), Barcelona, Spain; 3 CIBER Epidemiología y Salud Pública (CIBERESP), Barcelona, Spain; 4 Translational and Clinical Research Institute, Faculty of Medical Sciences, Newcastle University, Newcastle upon Tyne, UK; 5 National Institute for Health and Care Research (NIHR) Newcastle Biomedical Research Centre (BRC), Newcastle University and The Newcastle upon Tyne Hospitals NHS Foundation Trust, Newcastle upon Tyne, UK; 6 Research group on Methodology, Methods, Models and Outcomes of Health and Social Sciences (M3O), Faculty of Health Sciences and Welfare. Centre for Health and Social Care Research (CESS), University of Vic-Central University of Catalonia (UVic-UCC), Vic, Spain; 7 Institute for Research and Innovation in Life Sciences and Health in Central Catalonia (IRIS-CC), Vic, Spain; 8 Consorci de Salut i Social de Catalunya, Barcelona, Spain; 9 Department of Health Sciences, Lund University, Lund, Sweden; 10 Agència de Salut Pública de Barcelona (ASPB), Barcelona, Spain; 11 Department of Clinical Gerontology and Rehabilitation, Robert-Bosch Hospital, Stuttgart, Germany; 12 Geriatric Department, Heidelberg University Clinic, Heidelberg, Germany; 13 Federació d’Associacions de Gent Gran de Catalunya (FATEC), Barcelona, Spain; 14 Gerontology Research Center, Faculty of Sport and Health Sciences, University of Jyväskylä, Jyväskylä, Finland; 15 Wellbeing Services County of Central Finland, Jyväskylä, Finland; 16 Age-Friendly Environments Academy (AFEdemy), Gouda, The Netherlands; 17 ISGlobal - Dr. Aiguader 88, PRBB, Barcelona, 08003 Spain

**Keywords:** Aging, Walking, Physical activity, Environment

## Abstract

**Background:**

The aging of the world’s population and the increase in sedentary lifestyles are leading to an increase in walking impairments at older ages. Here, we aimed to comprehensively discuss walking in the context of an aging population; and identify and agree on a list of future research priorities and policy actions.

**Methods:**

We followed a participatory approach and held a multidisciplinary two-day workshop on October, 2023 in Barcelona, Spain, with experts in the fields of aging and walking, and participants from the general public.

**Results:**

A total of 56 national and international participants, from a multidisciplinary background, joined the workshop. They had a median age of 40 years (range 24–83), and 62% were female. Participants discussed the meaning of walking from different perspectives and its change with age and in the presence of diverse mobility-impairing conditions; the emotional and social components of walking; and the role of the environment in walking. Participants identified unmet needs, research priorities and policy actions related to walking in older ages.

**Conclusions:**

This two-day workshop provided a space for professionals and public to comprehensively discuss walking at older ages. Participants highlighted the relevance of a better and more comprehensive assessment of walking; the need to shift focus towards comprehensive health that considers physical, emotional and social aspects as well as individuals’ preferences and expectations; and the importance of translating research into action. Future work can draw on the discussions held during this event in a thought-provoking and hypothesis-generating way.

## Background

Walking is the most common type of physical activity and the simplest way to stay active and independent [1–3]. Walking can be performed for multiple purposes, such as engaging in basic and instrumental activities of daily living [2], as a mode of transportation [4, 5], or as a leisure time physical activity [1]. In all cases, walking reflects on individuals’ autonomy and ability to make decisions about their daily behavior, which has been highlighted as a paramount aspect of active ageing [6]. However, walking changes with age and in the presence of mobility-impairing health conditions (e.g., Parkinson’s disease, hip fracture) as individuals tend to slow down, walk less, and physical deconditioning occurs [7–9]. Moreover, global trends towards increased longevity coupled with an increasingly sedentary lifestyle have resulted in a higher prevalence of walking impairments in older adults, underpinned by a natural decline in functional capacity later in life [10–12]. This is further exacerbated in older adults living with mobility-impairing health conditions, and it relates to an increased risk of falling, disability, and mortality [7, 9, 13, 14].

While walking impairment and sedentarism pose a major risk to the health of the aging population, regular walking has proven to be an effective means to preserve cognitive and neural function [15], to manage pain and improve function in musculo-skeletal disorders [16], and to prevent physical and mental non-communicable diseases (NCDs) [16–19]. As a result, the World Health Organization (WHO) has included walking as a critical element of its strategy to promote Active and Healthy Aging (AHA) globally [17]. WHO strategic actions include: incentivizing physical activity for older adults, assessing data on walking and cycling activities, developing and disseminating benchmark recommendations on physical activity and sedentary behavior especially designed for aging populations (such as volume, type, and frequency) [19], and producing AHA toolkits for governments and the health sector [17]. Despite this huge global effort by WHO, current assessments (and resulting materials) are still incomplete, as they lack disaggregated data on walking, and guidance on the public open spaces where physical activity and walking at older ages can take place safely.

Traditional approaches to walking in older ages - and to interventions targeting it - have predominantly focused on specific components, such as the biomechanics of gait [20, 21], and the person-environment fit [22]. A substantial body of literature has examined the spatio-temporal, kinetic and kinematic characteristics of walking in later life and under mobility-impairing conditions [8, 20, 23, 24]. Similarly, extensive research has explored how older adults interact with environmental demands, examining the role of functional ability and of multi-dimensional aspects of the environment, such as the home and neighborhood characteristics, access to natural surroundings, and social cohesion and support within communities [25–32]. More recently, conceptual frameworks have shifted away from this component-specific approach, advocating for more comprehensive perspectives that characterize walking as a multi-faceted phenomenon [33, 34]. Notably, a recent meta-ethnography synthesizing walking experiences in the context of aging and mobility-impairing conditions, proposed a novel conceptual framework. This framework conceptualizes walking as a multi-faceted and dynamic experience, characterized by a constant interplay between physical, emotional, and social components, that are influenced by the (physical, social and personal) environment, the activities that individuals aim to perform, and the adaptive strategies they employ in response to behavioral changes [33]. However, applying such frameworks, and advancing a more holistic understanding of walking at older ages, requires interdisciplinary engagement. Researchers, healthcare providers, policymakers, and older adults themselves must be challenged to approach walking from multiple perspectives, fostering a more integrated and inclusive approach.

Taking all of this into consideration, the *Aging Towards Walkable Futures workshop* aimed to: (i) comprehensively discuss walking in the context of an aging population; and (ii) identify and agree on a list of future research priorities and policy actions. In this report, we present a synthesis of the main discussions held during the workshop and the key research and policy priorities we identified.

## Methods

### Participants

The workshop was held in Barcelona, Spain, on October 23–24, 2023. In collaboration with experts in the field of ageing and walking, we identified potential speakers and attendees in a purposeful manner. We aimed to: (i) include attendees from different background disciplines; (ii) cover diverse professional sectors; and (iii) being equitable with respect to representation of junior and senior speakers, gender balance, and involvement of vulnerable populations. We communicated directly with a list of candidate organizations, institutions and individual members, and we further advertised the event through ISGlobal and Mobilise-D social media and official websites. The workshop gathered 56 participants, including eleven speakers and three moderators. It brought together local and international attendees, 62% of whom were women, with a median age of 40 years (age range 24–83, Table 1). Participants included researchers, health professionals, policymakers, and citizen organizations, coming from different backgrounds such as medicine, public health, physiotherapy, urban planning, political science, and sports sciences. No ethics approval was required as the workshop does not fall into the biomedical research regulated by the Spanish Act 14/2007 of July 3. The workshop complied with national data protection regulations and attendees provided consent to the processing of their personal data for the purpose of communicating information related to the event.

**Table 1:** Distribution of 56 workshop participants according to gender, age, occupation and academic background

Demographic characteristics of workshop participants	All *n* = 56
Age (years), med (range)	40 (24–83)
Gender (female), n (%)	34 (62%)
Main occupation*, n (%)	
Health professional (geriatricians, other clinicians, physiotherapists, and nurses)	4 (7)
Policy (health policy, urban planning, civil society organizations)	21 (38)
Research (from public and private research centers and universities)	31 (55)
Academic background*, n (%)	
Applied sciences (engineering, urban planning)	7 (13)
Health sciences (medicine, biomedicine, physiotherapy, sport sciences)	37 (67)
Natural sciences (biology, mathematics)	3 (5)
Social Sciences (political science, philosophy)	8 (15)

### Workshop methodology

The workshop consisted of four sessions. Sessions 1 to 3 featured individual presentations focused on specific objectives: Session 1 explored ‘what walking means from different perspectives’ and ‘how walking changes with aging and in the presence of diverse mobility-impairing conditions’; Session 2 explored the ‘emotional and social components of walking for an aging population’; and Session 3 examined the ‘role of the environment for walking in older age’. Each session also included an hour-long round table discussion involving both the speakers and the audience, to identify key messages from each presentation and to address unmet research and implementation needs related to the specific objectives. Session 4, structured as a consensus-oriented discussion, built upon previous sessions’ discussions, and sought to summarize and identify research priorities and policy actions for the near future.

### Instruments to prepare for discussions and assess the impact of the workshop

**Fig. 1:**
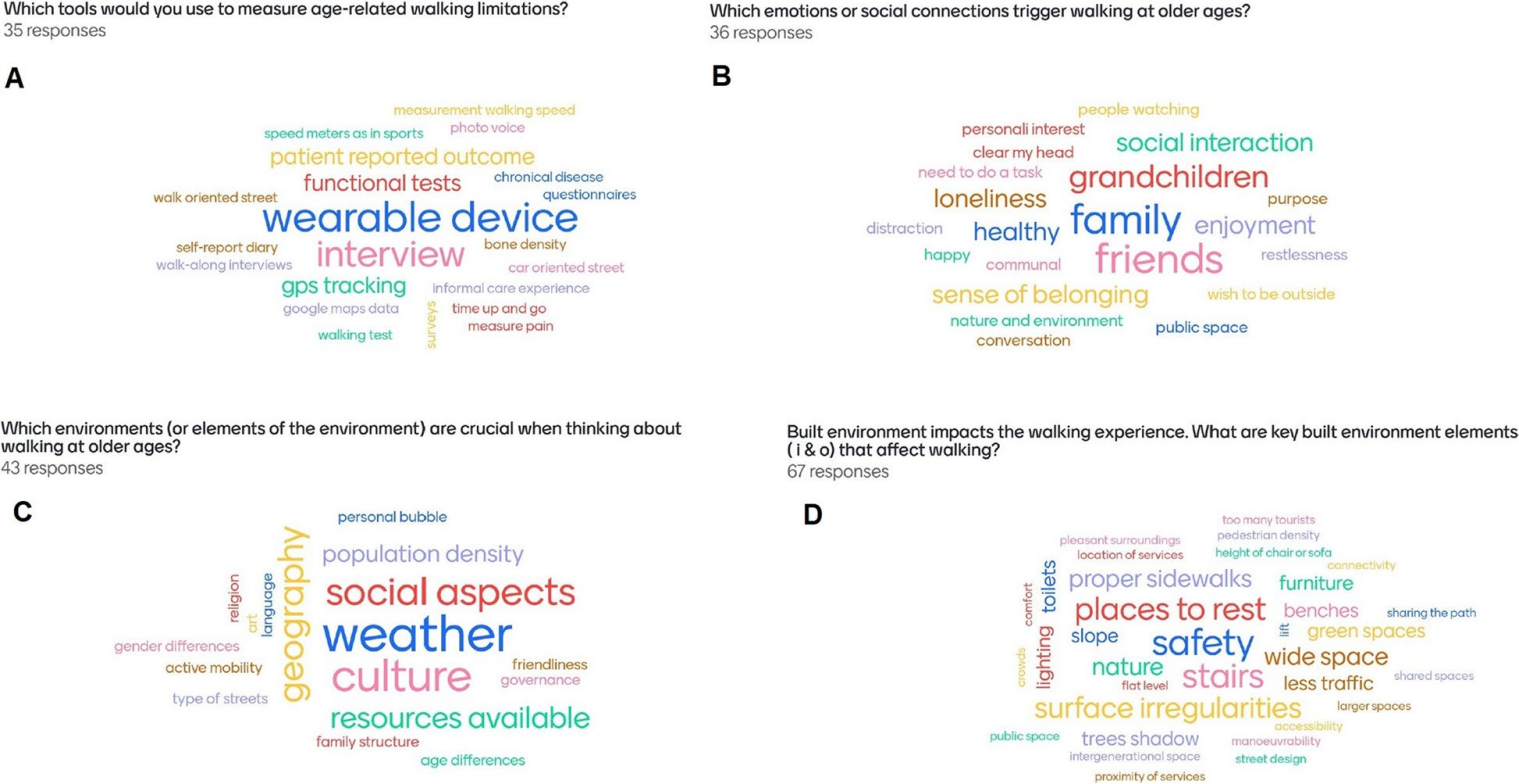
Instant assessments during the workshop Word clouds presented during the workshop discussions were developed using the Mentimeter platform and include anonymous replies from attendees

Before and after the workshop, we invited all registered participants, including speakers, moderators, and audience members, to complete two online questionnaires on workshop-related topics to assess the impact of the event on its participants. The pre-workshop questionnaire consisted of six Likert-scale questions assessing the key topics to be addressed in Sessions 1–3; and four questions assessing basic socio-demographic characteristics of participants (i.e., age, gender, background and main occupation). Following an interactive format, we used some of the pre-workshop responses to stimulate and guide discussion during the workshop, supplemented by instant assessments of the topics covered during the workshop (Fig. 1). The post-workshop questionnaire evaluated the impact of the event on participants, addressing changes in perspective, changes in personal experiences, opportunities to network, and identification of key research and policy priorities.

## Results

Fifty-six individuals (mean age 40 years (range 24–83), 62% women) registered in the two-day multidisciplinary *Aging towards walkable futures workshop* held in Barcelona (Table 1). A summary of the discussions held during this event is presented below, following the structure of its four thematic Sessions.

### Session 1. What is walking – a physical experience?

**Fig. 2:**
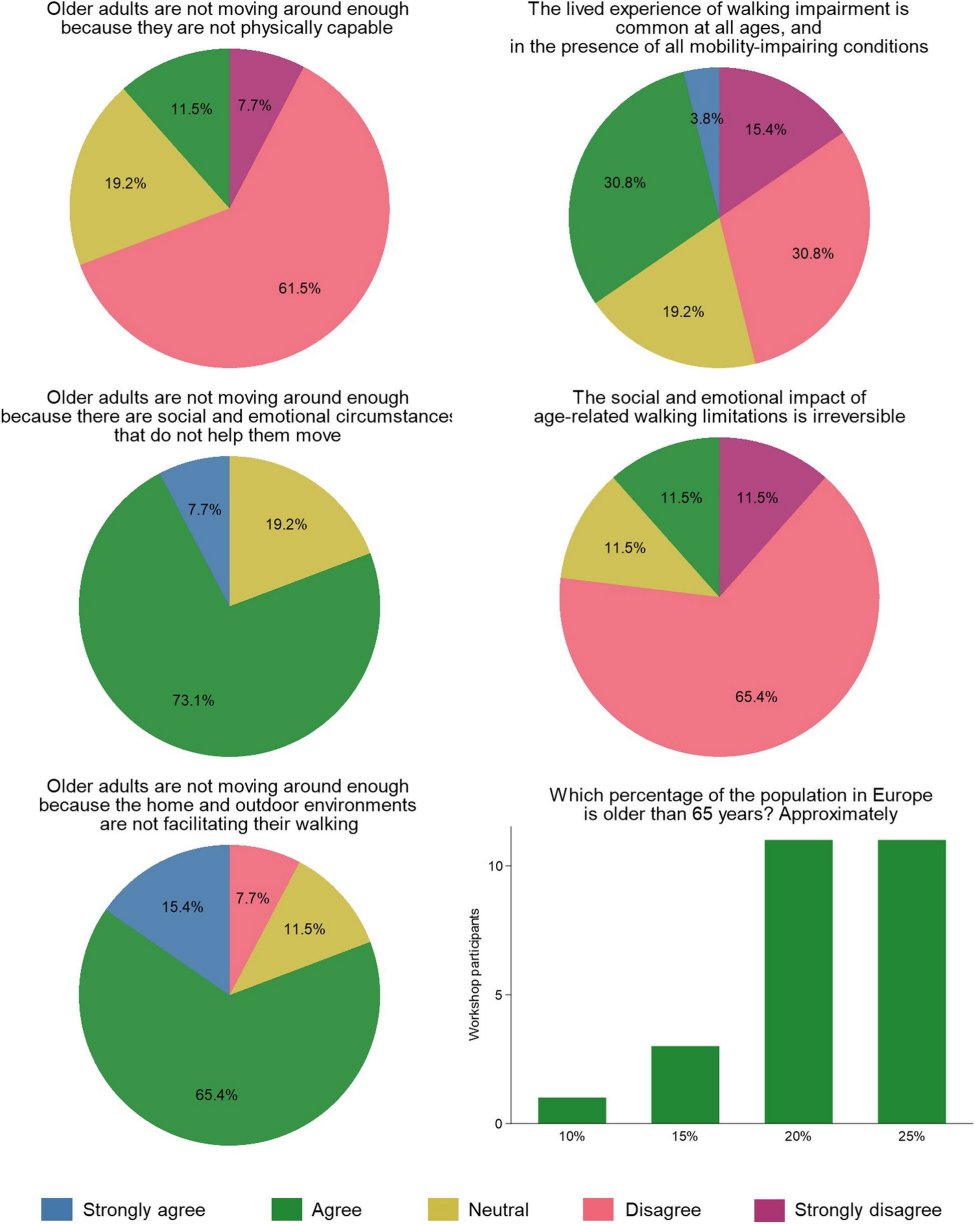
Assessment of workshop-related topics prior to the event Replies from the pre-workshop online questionnaire from 26 registered participants

Among participants who completed the pre-workshop questionnaire, 11.5% considered that older adults do not move enough due to physical incapacity, while 46.2% disagreed with the notion that walking impairments are experienced similarly across different ages and mobility-impairing conditions (Fig. 2).

The discussions in Session 1 built on these themes, revealing that walking is a complex physical activity, involving most of the physiological systems of the human body, including the nervous, cardiovascular, musculoskeletal, and respiratory systems. As we age, our walking capacity and quality deteriorates. As the overall levels of physical activity reduce, our walking speed decreases, physical deconditioning occurs, specific impairments related to health conditions appear, and the dependence on caregivers and/or assistive devices increases. Whilst we can mitigate some mobility losses through treatment and rehabilitation, others may require adaptation to a ‘new normal’.

Hence, assessing walking and walking impairment at older ages is of key importance to understanding what walking means in the context of aging populations. A growing body of research indicates that digital technologies (e.g., wearable sensors) are capable of measuring detailed characteristics of walking both in lab settings and in free-living scenarios (i.e., the real-world). Among these characteristics, walking speed stands out, as it has been proven as a valid and reliable parameter capable of predicting functional decline, hospitalizations, and mortality [7]. However, there are many more characteristics of walking, including the number of daily steps, cadence, or step length that can provide relevant information concerning the walking ability of older adults and individuals living with mobility-impairing conditions [35]. All of these walking parameters have the potential to be used in research and clinical settings to track functional decline, and disease progression, and to evaluate therapeutic responses. Recent developments and large-scale investigations on the technical validity and clinical application of sensor-derived characteristics of walking have pushed even further laboratory-based assessments to the real-world, thus improving the ecological validity of such data [36, 37].

Aside from the objective assessment of walking, a comprehensive understanding of what walking means in the aging population requires the consideration of additional perspectives that may be involved in the care continuum, including but not limited to healthcare professionals, healthcare systems and insurances, international organizations, caregivers, and older adults themselves. All of these perspectives are key to identifying and effectively treating walking impairments in older adults, as it can be seen in the following examples.

In the view of healthcare professionals, walking is often considered only as a physical capability. Thus, healthcare professionals assess whether individuals’ functional capacity is above or below a certain threshold, whether condition-specific signs and symptoms are being appropriately managed, or whether interventions like rehabilitation are leading to objective improvements. However, with this approach, other aspects of walking that are important to individuals are overlooked. For instance, while an older woman may be able to walk a long distance during a 6-minute walk test (i.e., in a clinical setting), and therefore considered physically fit enough for walking, she might be unable to walk the same distance while carrying her grocery bags, or while walking with her grandchildren around the neighborhood, tasks that may be more meaningful to her. Thus, a more comprehensive assessment of walking should consider not only individual’s physical health but also their personal needs, preferences and expectations.

The view of those representing healthcare systems and insurance companies is often restricted to evaluating costs and benefits of preventive actions that enhance active lifestyles. Indeed, walking has been proven to be a cost-effective way to achieve an AHA and to reduce healthcare costs globally [38]. However, this view is, again, limited, as some aspects of walking (e.g., those relevant to individuals) are often overlooked while performing cost-benefit analyses.

The view of older adults, and particularly of those living with mobility-impairing conditions, highlights walking as a complex experience, involving physical, emotional, and social components. When individuals are cognizant of changes in their walking, and the increased amount of effort required to perform the same tasks, walking becomes the link between the activities that individuals want to perform and their sense of self, something that challenges their identity and social roles, and prompts attitudinal and behavioral adaptations [33], in agreement with established theories describing the impact of disabling conditions on the need to constantly adapt and reconstruct [39, 40]. However, again, this view can be limited, in that individuals may lack the clinical expertise to qualify their functional capacity and act upon it, and they may disregard the future economic and social burden imposed by their decisions. Therefore, a holistic assessment of walking that takes into consideration all perspectives is needed.

Unmet needs identified in Session 1 are summarized in Table 2. During this session, we discussed how novel instruments that assess walking, walking impairment, and the personal experience of both are an unmet need that can be overcome by harnessing the advantages of digital technologies and knowledge from previous research (Fig. 1, panel a). This may prompt a shift in the current focus on functional capacity by healthcare professionals towards a more comprehensive approach that embraces a personalized assessment of walking and acknowledges the importance of the individual’s needs, preferences and expectations. Finally, and in agreement with previous calls from the WHO, we discussed on the current need to improve assessments by obtaining global data on public open spaces, and disaggregated data on the most common forms of locomotion, i.e., walking and cycling.

**Table 2:** Unmet needs for walking at older ages

**What is walking – a physical experience?** • Developing novel instruments to assess walking experience and walking impairment experience • Shifting the current focus from walking capacity towards a more comprehensive approach that acknowledges individual’s preferences and expectations • Improving worldwide data on walking and walking spaces **The emotional and social experiences of walking** • Including individual preferences and experiences in walking interventions • Targeting and tailoring walking programs for vulnerable aging groups • Incorporating the experiences and observations of caregivers more formally in the health-providing process • Providing specific resources for caregivers **Walkable environments for older adults** • Implementing research results on housing accessibility, walkability, and life-space mobility widely • Acknowledging cross-cultural and environmental differences while developing interventions in different life-spaces beyond the home • Informing multiple stakeholders on the importance of developing age-friendly environments and making recommendations for design and implementation	

This list arises from the discussions held in Session 1-4 of the Workshop

### Session 2. The emotional and social experiences of walking

Before the workshop, 80.8% of participants believed that older adults do not move enough due to emotional or social factors, but only 11.5% considered the impact of these factors on walking impairment to be irreversible (Fig. 2). The discussion in Session 2 reinforced these findings, emphasizing that for aging populations, emotional and social factors such as loneliness, presence of caregivers or other social support, and access to local organization activities, influence and are influenced by walking-related activities (Fig. 1, panel b).

Loneliness, a negative feeling related to the perception of a lack of company, is common in older adults [41] and is linked to social isolation and sedentary behavior [42]. Loneliness is a risk factor for adverse health-related outcomes [41], such as fatigue, cognitive impairment, and physical inactivity [43]. Nonetheless, loneliness ought to be distinguished from the positive feeling of solitude, which relates to being pleasantly alone with oneself. This distinction becomes particularly important when designing, developing, and promoting walking interventions. For instance, while walking groups are very popular, providing a source of peer support and a sense of belonging, solitary walks may be preferred by some older adults. Thus, considering the emotional and social experiences of walking, and individuals’ preferences is important when designing interventions, because tailored interventions that are enjoyed, are more likely to lead to behavior change and long-term maintenance.

Caregivers have an important influence on the emotional and social experience of walking at older ages. Formal and informal caregivers are key agents of clinical care, providing personalized assistance to older adults and enhancing their social engagement and walking-related activity; they often are the first to identify a change in walking and observe subtle changes due to continuous observation. However, many times they lack practical resources, support, and expertise to adequately carry out these tasks.

Having a social network, beyond caregivers, that is supportive of one’s health encourages individuals to stay active and walk more as they get older. More generally, being socially connected has a positive impact on physical and mental health [44, 45] and can be an important source of joy and fulfillment [46]. Thus, walking activities that trigger connections with friends, family, and caregivers may prompt positive behavioral change. Group-based walking consolidates and increases participation in social activities; and walking, whether alone or in a group, can elevate mood, reduce stress and anxiety, enhance confidence, and ensure better sleep routines.

Local and civil society organizations are also important contributors when it comes to considering the social and emotional experiences of walking at older ages, as they work to promote an AHA through group activities such as walking courses. An example was provided during the workshop by the *Federació d’ Associacions de la Gent Gran de Catalunya* (FATEC), an organization that reaches more than 450,000 older adults in Catalonia, Spain. The organization aims to improve quality of life through the promotion of active, healthy, and socially productive aging which in turn stimulates physical, mental, and social well-being and maintenance of personal autonomy [47]. FATEC’s work builds upon the idea that being socially connected has a positive effect on individuals’ emotional well-being.

Unmet needs identified during Session 2 are summarized in Table 2. Here, we discussed how interventions to promote walking in older adults could consider individual preferences and be further personalized to achieve the desired behavioral change. Tailored programs could be better integrated into healthcare systems and proactively prescribed in primary care. Vulnerable aging groups could be specially targeted by these programs, to promote health equity. We also identified a need to make better use of the experience of caregivers so that their insights can be used by primary care providers, clinicians and rehabilitators, and other healthcare providers. Additional resources for caregivers, such as educational programs and guidelines focusing on the identification of the walking impairment, should be provided to empower individuals and enable self-management.

### Session 3. Walkable environments for older adults

Before the workshop, 80.8% of participants believed that older adults do not move enough, due to lack of supportive home and outdoor environments (Fig. 2). Building on this perspective, the discussion in Session 3 highlighted that walking occurs in different life-spaces that can be classified according to their scope. Among these life-spaces, the home, neighborhood, and city have been largely studied by researchers in the fields of aging and walking (Fig. 1, panels c and d).

The home environment has a crucial role in the walking activity later in life. In the presence of age- or disease-related reduced functional capacity, the housing accessibility can be the determining factor between a sedentary, indoors life, or the flexibility to move around inside the home, and even go out outside onto the street. For instance, the presence of handrails in a building’s entrance or the presence of grabrails in the bathroom become important facilitators for individuals’ mobility. The example of the Housing Enabler was proposed as a research-based instrument that measures housing accessibility and quantifies accessibility problems as an important aspect of the person-environment fit [48, 49].

The neighborhood, as a broader life-space, has also been studied in the context of walking at older ages. The concept of walkability, defined as the extent to which the built environment is friendly to people who walk (and thus benefits the health of residents and increases the livability of built space) [50], appears most relevant in the study of this life-space. Walkable environments are easy to negotiate, accessible, compact, safe, and physically enticing (i.e., rich in pedestrian-oriented infrastructure), and may result in lively and sociable, exercise-inducing spaces, ideally supplied with sustainable transport options [51]. Research shows that living in highly walkable areas is associated with higher participation in cultural activities [52], which in turn increases walking activity at older ages. Existing research also shows that walking in neighborhoods increases with more perceived environmental facilitators (e.g., access to green areas, attractive features in the nearby environment) [29], whilst it decreases with more perceived environmental barriers (e.g., long distances to services, uneven surfaces, or insecurity) [53], which suggests that the perception of one’s environment has the power to constrain or extend livable spaces at older ages [54]. Importantly, neighborhood barriers can be addressed by either environmental modifications (e.g., improvements in urban fabric) or walking adaptations (e.g., slowing down, pacing) [27, 55].

Moreover, it is important to recognize that the effect of neighborhood characteristics on walking is highly context-dependent. For instance, while cities like Barcelona (Mediterranean coast in Spain) or Jyväskylä (Central Finland) are known to have highly walkable neighborhoods in which older people may perform higher amounts of physical activity on broader life-space areas [28, 30], their specific contexts (e.g., culture, weather) may have an impact on the walking adaptations assumed by older residents. As an example, in Jyväskylä, a city characterized by the cold weather and snow that lasts for up to six months per year, outdoor physical activities of older adults are limited, and walking needs to be facilitated by modes of motorized transport (e.g., cars, buses) [28, 29, 53], so individuals can reach large indoor open spaces where they can walk, such as shopping malls.

Beyond the neighborhood, the city can encourage active aging by optimizing opportunities for health, participation, and security, as proposed by WHO [56]. New concepts, such as Age-Friendly Cities and Smart Healthy Age-Friendly Environments (SHAFE), study the impact of cities on walking at older ages. From a practical perspective, an Age-Friendly City is a city that adapts its structures and services to be accessible to and inclusive of older people with varying needs and capacities [56]. The second concept, SHAFE, incorporates the phenomenon of digitalization into the concept of Age-Friendly Cities [57]. The way cities are becoming age-friendly and facilitate walking in the case of older adults and other citizens is gaining increasing attention from different stakeholders, including, but not limited to, academics, policymakers, urban planners, and the private construction industry; all of which need to work in collaboration in the current context of urbanization and growing aging population.

During the workshop, the example of a public health intervention designed in Barcelona to promote physical activity among older adults was discussed. In the context of the Health in Neighborhood’s Program, the *Activa’t als parcs (‘Get active in parks)* intervention provides group-based physical activities in public parks of Barcelona and reaches over 1,700 older adults [58]. Specific characteristics of the physical environment such as pleasant shady areas, benches, and separate lanes for pedestrians and bicycles, are important walking enablers for older adults participating in these group-based activities. Likewise, highways or lack of adequate infrastructure hinder the participation and adherence of older adults to these types of programs. Interventions like *Activa’t als parcs* ought to further consider the sociodemographic characteristics of their target population, to guarantee their appropriateness and effectiveness. As such, a context- and population-specific approach seems to be absolutely necessary.

Unmet needs identified during Session 3 are summarized in Table 2. During this session, we discussed how housing accessibility, walkability, and life-space mobility have been widely explored and covered through research. Despite this, there is a growing need for implementing the knowledge generated to enhance walking later in life. In addition, there is a need to communicate with multiple stakeholders, including policymakers and building planners, on the importance of developing age-friendly environments and the potential of initiatives such as SHAFE.

### Session 4. Setting the future of walking for older adults

Our comprehensive discussion of walking at older ages resulted in a reflection of not only its physical, emotional, and social aspects, but also of the role of the environment where walking occurs, and the multiple stakeholders that should be considered. Walking at an older age is a multi-component construct and there is a need to understand its complexity. Discussions on this construct can be tailored to different aspects such as [1] walking capacity and performance [2], walking experience [3], walking impairment, and [4] walking impairment experience. The assessment of each one of these aspects requires specific instruments, that may already exist and need to be revisited or escalated (e.g., wearable sensors to assess real-world walking), or that may need to be developed and validated (e.g., hybrid tools to assess walking impairment experience). Hence, there is a need to keep advancing the comprehensive assessment of walking at older ages with novel instruments and parameters to monitor walking status, predict health outcomes, and investigate the role of the person-environment interactions and how they impact walking (Table 3).

**Table 3:** Identified research priorities and policy actions for walking in older ages

**1. Better assessment** • Comprehensive assessment of walking and walking impairment • Development and/or implementation of targeted tools • Use of digital technology to enhance the assessment and monitoring of walking in lab and real-world settings • Global disaggregated data on walking **2. Shift in focus** • From physical health to comprehensive health, including social and emotional aspects, as well as the person-environment fit • Moving away from the ‘one size fits all’ approach: development of tailored programs to enhance walking at older ages, considering individuals’ preferences and expectations. • Addressing ageism **3. Implementation of research results** • Implementation of research on walking for older adults at local and international levels • Development of walking-enhancing strategies that consider the key characteristics of the environment where they are being implemented • Collaboration of researchers with key stakeholders	

This list arises from the discussions held in Session 1-4 of the Workshop

Although more research into the effects of regular walking and walking impairment on health outcomes will always be beneficial, especially in the case of novel conditions such as long-COVID, there is already a fairly good understanding of how walking is impacted by mobility-impairing health conditions. Hence, there is a need to shift the focus of health evaluation and treatment from physical health to a comprehensive assessment of walking, considering also important social and emotional aspects such as expectations and preferences, life satisfaction, social role fulfillment, and enjoyment (Table 3). Moreover, it is important to address ageism in academic research as well as in everyday life. Using the correct terminology and confronting negative stereotypes is a pending task in many disciplines including gerontology.

Finally, there is a fundamental need to offer programs and interventions that increase the well-being of the aging population by increasing and maintaining walking activity. An implementation gap currently exists between what is already known in academia and what is being implemented by different stakeholders at national and international levels (Table 3). Thus, researchers should work closely with other stakeholders, to inform and educate them, improve the assessment and treatment of walking and walking impairment, make environmental modifications that promote walking and, ultimately, promote walking at older ages.

### Impact assessment

We evaluated the impact of the event through a post-workshop questionnaire, answered by 13 participants. The results revealed that 76.9% of participants reported a shift in their perspectives of walking in older adults as a result of the discussions held during the event. Additionally, 61.5% reported changes in how they personally experience walking, and 92.3% agreed that the event provided opportunities to network and engage with diverse stakeholders working in the field (Table 4).

**Table 4:** Impact assessment from the post-workshop online questionnaire

Impact assessment	All (*n* = 13)
Have your perspectives on walking in older adults changed based on what you have learned (yes), n (%)	10 (76.9)
Does this workshop change the way you will experience walking (yes), n (%)	8 (61.5)
The workshop provided you with opportunities to network with others working in the field of walking at older ages, and to engage with different stakeholders Strongly disagree Disagree Neutral Agree Strongly agree	1 (7.7) 0 (0.0) 0 (0.0) 8 (61.5) 4 (30.8)

## Discussion

The *Aging Towards Walkable Futures workshop* provided a platform for diverse stakeholders to engage in discussions about walking at older ages. The event built upon existing conceptualizations of walking as a multi-component phenomenon in the context of aging and mobility-impairing conditions [33, 34], and reached consensus on the need for a comprehensive health approach addressing the physical, emotional, social and environmental aspects of walking. Participants also emphasized the importance of developing more comprehensive assessments of walking and walking impairment.

The interdisciplinary nature of the event, reflected in the variety of disciplines represented, fostered rich exchanges and contributed to a more holistic understanding of walking in later life. For instance, Fig. 1, and the examples from Jyväskylä and Barcelona, illustrate the range of perspectives that shaped discussions during the event. Interestingly, the gap between scientific knowledge and its implementation at the local and international levels, was openly discussed by participants during Session 4, reinforcing the value of workshops like this one, where researchers were able to engage with policymakers to explore collaborative avenues. In this sense, results from our impact assessment also indicated a positive evaluation of the event and its networking opportunities.

Our findings reflect insights from an experts’ review conducted during a two-day workshop. Strengths of this method are: first, its multidisciplinary approach to discuss walking at older ages, allowing us to gather researchers, healthcare professionals, policymakers and relevant stakeholders from civil society organizations and collectively discuss on the same topic; and second, the inclusion of participants from diverse geographical locations, enriching discussions with different contexts and lived experiences. However, our approach also has a few shortcomings. First, the purposive selection of speakers done in collaboration with experts in the field, may limit the generalizability of our findings. Second, while we aimed to identify concrete priorities for future research and policy actions, the identified avenues for future work are limited to the discussions held during the event, and may not be comprehensive enough of all the additional work required in this field. Third, while we actively invited older adults to join the event, we had a relatively small subgroup of individuals > 60 years, although they contributed with their valuable lived experiences to discussions. Finally, while we aimed to evaluate the impact of the event on all its participants, only 23% of them responded our post-workshop online questionnaire, limiting the generalizability of impact results and introducing potential bias; future research should explore alternative formats to better assess impact of similar events.

## Conclusions

Walking at an older age is a multi-component construct that incorporates physical, emotional, and social aspects which in turn are influenced by the environment. Ongoing work in this field is multidisciplinary, but needs to be integrated to better address the complexity of walking in later life. The workshop *Aging Towards Walkable Futures* provided a space for professionals and public from different backgrounds to comprehensively discuss walking in the context of an aging population and to identify and agree on a list of unmet research, healthcare, and policy needs. Participants highlighted the relevance of a better and more comprehensive assessment of walking; the need to shift focus towards comprehensive health that considers physical, emotional and social aspects as well as individuals’ preferences and expectations; and the importance of translating research into action. Future work can draw on these discussions in a thought-provoking and hypothesis-generating way.

## Data Availability

All data generated or analyzed during this study are included in this published article.

## Abbreviations

AHA: Active and Healthy Aging
FATEC: Federació d’ Associacions de la Gent Gran de Catalunya
NCDs: Non-communicable diseases
SHAFE: Smart Healthy Age-Friendly Environments
WHO: World Health Organization
